# Supplementary material for: SARS-CoV-2 Neutralizing Antibodies to B.1 and to BA.5 Variant after Booster Dose of BNT162b2 Vaccine in HIV Patients COVID-Naïve and on Successful Antiretroviral Therapy
Source: Vaccines (Basel). 2023 Apr 20;11(4):871. doi: 10.3390/vaccines11040871 (PMC10144758; doi:10.3390/vaccines11040871)
Supplement: Supplementary file 1 [file vaccines-11-00871-s001.zip › SG Parisi Supplementary Table S1 230323.pdf]

Table S1. Neutralizing antibody titers to B.1 and to BA.5 classified as outliers (n=19). Data are expressed as ID_{50}: reciprocal value of the sample dilution that showed a 50% protection from the virus-induced cytopathic effect.

	N (%)	Median ID_{50} (IQR)
Outliers only to B.1	2 (10.5)	3139 (2541-3738)
Outliers only to BA.5	5 (26.3)	495 (445-1196)
Outliers to B.1 in patients with outliers to both strains	6 (31.6)	3723 (2730-4638)
Outliers to BA.5 in patients with outliers to both strains	6 (31.6)	901 (861-1024)
