# Supplementary material for: SARS-CoV-2 Neutralizing Antibodies to B.1 and to BA.5 Variant after Booster Dose of BNT162b2 Vaccine in HIV Patients COVID-Naïve and on Successful Antiretroviral Therapy
Source: Vaccines (Basel). 2023 Apr 20;11(4):871. doi: 10.3390/vaccines11040871 (PMC10144758; doi:10.3390/vaccines11040871)
Supplement: Supplementary file 1 [file vaccines-11-00871-s001.zip › SG Parisi Supplementary Table S2 230323.pdf]

Table S2. Raw data and comparison after outliers (n=13) removal.

**Test\parameter**	**Raw data (n=100)**	**Sensitivity analysis (n=87)**
**Correlation analysis**		
**Pearson's r**	0.56	0.69
**p-value**	< 0.001	< 0.001
*** R^2**	0.31	0.48
*** (df model; df residual) F**	(1;98) 44.48	(1;85) 77.79
*** p-value**	< 0.0001	< 0.0001
*** Coeff.**	0.18 (0.13 – 0.23)	0.12 (0.09 – 0.14)

*: Parameters relative to univariate linear regression model; df: degrees of freedom
